# A Simplified Sanger Sequencing Method for Detection of Relevant SARS-CoV-2 Variants

**DOI:** 10.3390/diagnostics12112609

**Published:** 2022-10-27

**Authors:** Felice Deminco, Sara N. Vaz, Daniele S. Santana, Celia Pedroso, Jean Tadeu, Andreas Stoecker, Sueli M. Vieira, Eduardo Netto, Carlos Brites

**Affiliations:** Laboratório de Pesquisa em Infectologia, Hospital Universitário Professor Edgard Santos, Universidade Federal da Bahia (UFBA)/EBSERH, Salvador 40170-110, Bahia, Brazil

**Keywords:** SARS-CoV-2, Sanger, variants, surveillance

## Abstract

Molecular surveillance of the new coronavirus through new genomic sequencing technologies revealed the circulation of important variants of SARS-CoV-2. Sanger sequencing has been useful in identifying important variants of SARS-CoV-2 without the need for whole-genome sequencing. A sequencing protocol was constructed to cover a region of 1000 base pairs, from a 1120 bp product generated after a two-step RT-PCR assay in samples positive for SARS-CoV-2. Consensus sequence construction and mutation identification were performed. Of all 103 samples sequenced, 69 contained relevant variants represented by 20 BA.1, 13 delta, 22 gamma, and 14 zeta, identified between June 2020 and February 2022. All sequences found were aligned with representative sequences of the variants. Using the Sanger sequencing methodology, we were able to develop a more accessible protocol to assist viral surveillance with a more accessible platform.

## 1. Introduction

SARS-CoV-2, a new coronavirus that causes a severe acute respiratory syndrome (COVID-19), was first reported in December 2019 [1] and soon after became a pandemic due to its high transmission rate, which favored the formation of new genomic mutations and contributed to the emergence of new variants [2].

As of November 2020, three new variants of the coronavirus emerged, originating from Brazil (gamma variants -P.1), the United Kingdom (alpha variant -B.1.1.7), and South Africa (beta varian-B.1.351) [3,4,5]. They rapidly spread across continents and were classified by the World Health Organization (WHO) as Variants of World Concern (VOC). Currently, the sub-variants of omicron (B.1.1.529), BA.2, BA.4, and BA.5, are the only ones with this classification [6].

In the middle of 2021, the delta variant caused an outbreak, becoming the most prevalent and worrying worldwide [7]. However, the emergence of new cases and the prevalence of the omicron variant in South Africa evidenced the change in the epidemiological landscape, whereas the circulation of the delta variant has been suppressed by the high transmissibility of the omicron [8].

Molecular surveillance of the new coronavirus through new genomic sequencing technologies has played a fundamental role in the monitoring of COVID-19 evolution by allowing the identification and screening of the main variants responsible for outbreaks around the world [9].

Methodologies involving Next Generation Sequencing (NGS) has been essential during the pandemic, from the detection of the etiological agent in the first samples until the determination of variants from the sequencing data deposited on platforms such as GISAID [10]. However, NGS is a platform with restricted accessibility due to operational costs, making difficult the viral molecular tracking in a widespread way [11]. Although Sanger sequencing is an old methodology, it is still very useful for mutation analysis and confirmation of NGS data. Recently, it has been used to identify important variants of SARS-CoV-2 without need of the whole-genome sequencing [11].

In this study, we implemented an amplification and sequencing protocol for SARS-CoV-2 to identify the most important variants from a segment of the S gene. Our protocol included the Receptor Domain Binding (RBD) in the S1 subunit, the S1/S2 cleavage site, and the start of the S2 subunit, thus spanning amino acids K417 through T716, in a single 1120 bp PCR product.

## 2. Materials and Methods

### 2.1. Study Design

This is an observational cross-sectional study, developed at the Research Laboratory for Infectious Diseases (LAPI) at Hospital Universitario Professor Edgard Santos (HUPES) in Federal University of Bahia, Salvador, Brazil.

We selected preserved samples, positive for SARS-CoV-2, from individuals over 18 years of age, who came to this center with symptoms of infection, as well as from HUPES healthcare workers who developed COVID-19. Individuals were attended to at the HUPES pneumology outpatient clinic from June 2020 to February 2022. All samples from involved participants were confirmed for COVID-19 through [12] detection of SARS-CoV-2 in saliva samples by an RT-qPCR assay, using the commercial kit 1copyTM COVID-19 qPCR 4plex Kit (1Drop, Seongnam-si), for amplification of 3 genes: E, N, and RdRp. Samples were considered detectable with Cycle Threshold (C.T) equal to or less than 40 in all 3 genes.

### 2.2. Amplification Protocol

We used 2.5 µL of RNA extracted from saliva in a reverse transcription assay using the SuperScript III Reverse Transcriptase kit (Invitrogen, Waltham, MA, USA) according to the manufacturer’s manual. The cycles of the two stages are 72 °C at 5 min (1st stage), thermal shock at 3 min (after thermal time), 50 °C at 10 min, 55 °C at 10 min, 58 °C at 58 min, and 94 °C at 1 min (2nd stage). For PCR reaction, 6 µL of reverse transcriptase reaction was used at a final volume of 25 µL. The enzyme used was Platinum Taq Polymerase from Brazil (Invitrogen, Massachusetts). The sequences of primers for the first and second rounds of amplification, shown in Table 1, were taken from published works [13] (https://github.com/artic-network/artic-ncov2019/blob/master/primer_schemes/nCoV-2019/V3/nCoV-2019.tsv accessed on 12 April 2021). The concentrations of all reagents used followed the guidelines of the enzymatic kits. The cycling of the first round is 94 °C for 2 min, 94 °C for 20 s, 60 °C for 30 s, 72 °C for 1 min and 30 s for 35 cycles, and 72 °C for 2 min. An amount of 2 uL of the first round was used for a second round PCR (Nested PCR) in a final volume of 60 uL, cycling at 94 °C for 2 min, 94 °C for 20 s, 60 °C for 30 s, 72 °C for 1 min and 10 s for 35 cycles and 72° for 2 min. At the end of the PCR, 10 uL of the product was used for electrophoresis in a 1.5% agarose gel.

### 2.3. Purifications and Sequencing

We used the commercial kit PureLink Genomic DNA Mini Kit (Invitrogen, MA, USA) according to the manufacturer’s manual, obtaining a purified product of 40 uL. The sequencing primers can cover a region of about 1000 base pairs (bp) in the S gene. The sequencing reaction was performed using the BigDye Terminator V3.1 Cycle Sequencing kit. The BigDye reaction product was purified using 75% isopropanol, and at the end 10 uL of formamide was added to each well for sequencing using the SeqStudio (Applied Biosystems, Waltham, MA, USA).

### 2.4. Data Analysis

The results were analyzed by the Geneious 9.0.5 software (Dotmatics, MA, USA) for the construction of the consensus sequence from the reference sequence NC_045512.2 and identification of mutations along the genome. Sequences that contained mutations common to important variants were aligned to others, deposited on the GISAID and NCBI GenBank platforms, representative of variants of concern. The sequences generated in this study were deposited on the GISAID and GenBank platforms’ Table S1. The classification adopted for the variants was based on the PANGO lineage nomenclature, proposed by Rambout et al. 2020 [14].

## 3. Results

**Figure 1:**
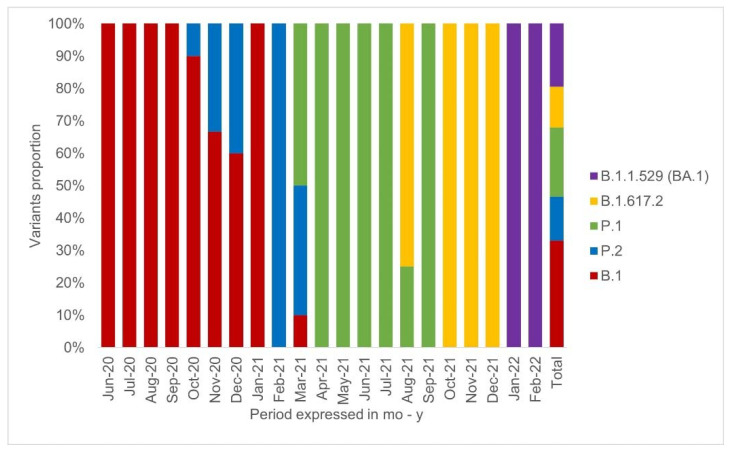
Distribution of studied variants in 2020 to 2022. Distribution of the variants detected in the study period, by month: wild type (B.1) as red, zeta (P.2) as blue, gamma (P.1) as green, delta (B.1.617.2) as yellow, omicron (BA.1) as purple. It can be observed that the B.1 strains and the P.1 variant had the highest prevalence among the analyzed samples, while the BA.1 was the only variant found in the first two months of 2022.

A total of 103 samples obtained from people with COVID in the period from 23 June 2020 to 12 February 2022 were used (Figure 1). The proportion of men in the sample was 53.2%. The age of the participants ranged from 18 to 85 years with a mean of 48 years. Among all the participants, cough was the most prevalent symptom (60%); HIV infection was the most common health condition, comprising 18% of the samples collected.

**Figure 2:**
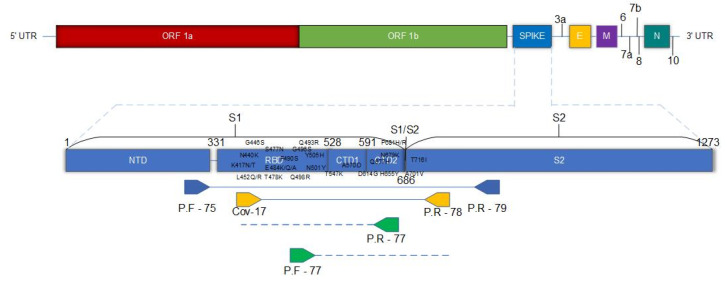
Primer placement and detection coverage. Positioning of the amplification and sequencing primers within the S gene. The positioning of the primers allows identification of the 25 mutations signaled in the image. The first round of PCR (primer forward 75 and primer reverse 79) amplifies an extensive region of the gene, covering the entire portion of RBD up to the initial portion of the S2 subunit. The product of the second round (Primer Forward CoV-17 and Primer Reverse 78) delimits the region for sequencing and identification of mutations.

We were able to amplify a single, extensive region of DNA of 1120 bp in all samples, which is enough to identify important mutations for the characterization and differentiation of emerging variants (Figure 2). The CT values for the samples ranged from 4.6 to 37.

In total, 28 important mutations are potentially identified, all located between amino acids K417 to T716 that are present in VOCs and VOIs, from position 1251 to 2148 of the S gene (Table 2). In all samples, we identified 23 mutations. All of these mutations allowed us to identify and differentiate 15 viral variants, including 4 omicron subvariants.

Among the sequenced samples, we found 4 relevant variants, represented by 13 delta variants (19%), 20 omicron variants (BA.1) (29%), 22 gamma variants (32%), and 14 zeta variants (20%) in a total of 69 samples. Currently, BA.2, BA.4, and BA.5 are classified as VOC. Two samples presented mutations suggestive for Lambda (C.37), L452Q and D614G, but none showed the F490S mutation. Thirty-four samples (33%) belong to the wild lineages (B.1) that prevailed at the beginning of the pandemic.

**Figure 3:**
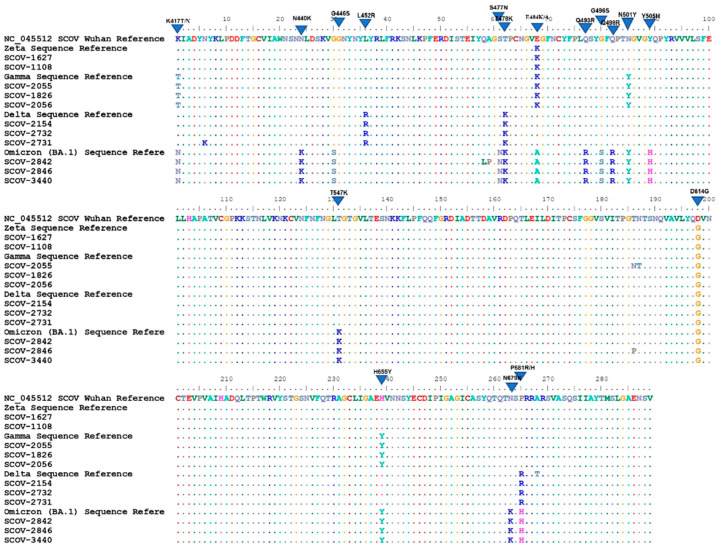
Alignment of the sequences with the representative of each variant and the reference generated in Wuhan. Protein sequences of some samples were aligned with templates for zeta (P.2), gamma (P.1), delta (B.1.617.2), and omicron (B.1.1.529). BA.1 plus the reference sequence generated in Wuhan. This alignment was done in the BioEdit program. The mutation profile found in each sample was paired with the one found in the template samples, confirming the classification adopted for the samples (SCOV-1627, SCOV-1108—zeta; SCOV-2055, SCOV-1826, SCOV-2056—gamma; SCOV-2154, SCOV-2732, SCOV-2731—delta; SCOV-2842, SCOV-2846, SCOV-3440—omicron). The illustrated protein alignment corresponds to position 417 to 705 of the spike.

The sequences corresponding to the variants found were aligned with representative sequences of these variants, deposited on the GISAID platform, plus the first one generated in Wuhan, NC_045512.2. All detected mutations representative of each variant were paired with those found in the sequences removed for alignment (Figure 3).

## 4. Discussion

The emergence of new SARS-CoV-2 variants has raised concerns about the new challenges of the pandemic. Sanger sequencing seems to be a viable alternative tool for identifying emerging SARS-CoV-2 variants [15]. In this work, we used Sanger sequencing technology to construct a simplified protocol for detecting the most important variants in the global scenery. We were able to identify several variants of SARS-CoV-2 during two years of pandemic, using a simplified protocol that allowed us to follow the changes in circulating variants.

The protocol was constructed with the aim of identifying 28 key mutations in the S gene of SARS-CoV-2, RBD, S1/S2 cleavage region and the beginning of the S2 subunit. In this region there are mutations characteristic of 11 main variants of the coronavirus plus 4 subvariants of omicron (B.1.1.529), such as Alpha (B.1.1.7), Gamma (P.1), Delta (B.1.617.2), BA.1, BA.2, BA.4, and BA.5 (B.1.1.529), Beta (B.1.351), Zeta (P.2), Theta (P.3), Lambda (C.37), Eta (B.1.525), Iota (B.1.526), Kappa (B.1.617.1), Mu (B.1.621), and Epsilon (B.1.427) [2].

Other authors using the same technology were also able to identify mutations in the same region [16,17,18]. Salles et al. developed a sequencing protocol covering the entire S gene where they were able to identify the gamma variant without the need to sequence the entire genome [11]. Besides the gamma variant, others such as delta, alpha, beta, epsilon, iota, kappa, and eta could be identified by other authors by sequencing five regions of the S gene [19]. Our protocol has the advantage of identifying the main variants of the coronavirus using only a single amplified fragment.

NGS platforms are useful tools for tracking viral gene variability in an infected individual, as well as for tracking mutations not detected by the Sanger platform [20]. As the pandemic progressed and new variants emerged, the genome sequencing data could not timely follow the increase in the number of cases worldwide, as more than 600 million cases were reported by May 2022 and only 13 million sequences have been deposited in the GISAID database since the beginning of the pandemic [9]. This confirms the weakness of current viral molecular surveillance, as the use of these platforms is concentrated in places with more sophisticated facilities and greater research resources. In addition, for a virus such as SARS-CoV-2 that does not present a high mutation rate [21] observed in other viruses such as HIV [22], Sanger technology becomes a more affordable alternative for screening important variants associated with new outbreaks worldwide [15].

In our study, we were able to sequence 103 samples from different periods of the pandemic, distributed over the peak periods. There were 38 (37%) between June 2020 and December 2020, 45 (44%) during year of 2021, and 20 (19%) in the months of January and February 2022. The C.T value of all samples was used to evaluate the effectiveness of the protocol against different viral loads. Our protocol was able to sequence samples with C.T from 4.6 to 37.0, showing its robustness despite the variation of viral load in clinical samples.

We included samples from 2020 to 2022, which provided us a way to follow the prevalence of variants over time. We detected the circulation of four important variants in addition to those belonging to wild strains predominant in 2020. In Bahia, in June 2020, variants B.1.1.28 and B.1.1.33 dominated the epidemiological picture, being suppressed by the emergence of gamma (P.1) and zeta (P.2) in January 2021 [23]. In total, we identified 34 samples containing wild-type strains, not specified by our protocol.

Among the important variants found in 69 samples, four variants are no longer circulating: gamma (P.1), zeta (P.2), delta (B.1.617.2), and the omicron subvariant BA.1 (B.1.1.529). When it was discovered, the omicron variant had 36 spike mutations, 15 of which are in RBD, some of which are present in other variants such as delta, beta, and gamma (K417N, T478K, N501Y, H655Y and P681H) [24] and are associated with viral escape to vaccines [25,26]. Our protocol was able to identify 13 mutations belonging to this variant, in which it was possible to visualize omicron-specific mutations (N440K, G446S, S477N, E484A, Q493R, G496S, Q498R, Y505H, T547K, N679K) [24,27].

The omicron variant is composed of several sublines, three of which are considered VOC, BA.2, BA.4, and BA.5, and basically have the same number of mutations [28,29]. Currently, BA.4 and BA.5 dominate the world epidemiological scenario, demonstrating greater transmissibility than BA.2. In addition, BA.4 and BA.5 can reinfect individuals who have already had COVID-19 by previous subvariants of omicron [30,31]. In our study, we identified 20 omicron variants, all characterized as BA.1. We did not identify the newest subvariants. Our protocol can differentiate BA.1, BA.2, BA.4, and BA.5, even though it cannot cover mutations such as D405N and D408S present in BA.2, BA.4, and BA.5. It is also able to continue identifying relevant variants as the viral evolution progresses. The absence of substitutions G446S, G496S, and T547K in BA.2 and the presence of L452R, F486V in BA.4/BA.5 became a differentiating criterion between them (Table 2) [27,28].

Thirteen sequences were classified as delta variants. This variant was first discovered in October 2020 but named by the WHO in May 2021 [6]. It has been considered a VOC due to its high transmissibility, short incubation period, and potential evasion of the neutralizing activity of antibodies generated by vaccine and previous infections [7,32]. In these samples, we identified three delta-specific mutations (L452R, T478K, and P681R) in addition to D614G, in agreement with other sequencing protocols, that used the same technology, capable of identifying delta [33].

The gamma variant, which is no longer important in the overall epidemiological picture of the pandemic, was identified in 22 samples. This variant was initially discovered in Japanese travelers returning from Manaus in November 2020 and was associated with the peak of cases in that city [34]. In these samples, we could find the common mutations described to gamma (K417T, E484K, N501Y, and H655Y). The E484K mutation was also found in 14 more samples, identified as zeta, a former VOI that is no longer circulating. Zeta was prevalent in Brazil in late 2020, where its emergence was accompanied by the spread of strains containing the E484K mutation [35].

The target region of our sequencing protocol comprises important amino acids for the infection mechanism of SARS-CoV-2 [36]. The amino acid K417, the first to be identified in our protocol, is part of the receptor-binding domain and is immediately adjacent to the receptor-binding motif (RBM), which encompasses the amino acids at positions 438 to 506 [2]. The S1 domain exhibits greater variability in amino acids compared to the S2 domain, with RBM being the most variable portion of RBD [2]. Within this RBM region, there are numerous important mutations, and all the VOCs already identified have at least one mutation in this region, conferring resistance to neutralizing antibody action and enhanced antigen-receptor interaction [37,38,39,40]. The enzymatic cleavage region between S1 and S2 stands out, because three essential mutations for the identification of important variants (H655Y, N679K and P681H) increase the proteolytic activity in this region, leading to increased viral infectivity and replication [27].

Mutations in the S gene responsible for the genotypic characterization of SARS-CoV-2 variants also influence their phenotypic characteristics, conferring escape to antibodies generated by vaccine or previous infections. Booster doses have emerged with the goal of improving vaccination coverage due to the emergence of new variants and the possibility of reinfection [41,42]. Success in identifying the variants through our protocol allowed us to observe that 100% of those who had the omicron variant and 70% of those who became infected with delta were vaccinated. The current protocol proved to be a simplified and effective tool for variant detection and could be applied to variant monitoring in vaccinated individuals.

## 5. Conclusions

Molecular surveillance of SARS-CoV using genome sequencing methodologies has provided worldwide data on viral evolution and adaptability as new cases emerged. The use of NGS for this purpose played an important role in this pandemic, although it is a platform of limited access due to operational costs. With the Sanger sequencing methodology, we were able to develop a more accessible protocol, which allowed us to identify four important variants from a single amplified DNA fragment. Thus, this protocol can assist viral surveillance from a more accessible platform.

## Figures and Tables

**Table 1:** Information of primers used in nested PCR and sequencing.

	Primer’s Name	Sequence	T.M	T.A	Position
**1R**	Primer foward 75 ^(a)^	AGAGTCCAACCAACAGAATCTATTGT	64.5°	60°	22.517–22.524
	Primer reverso 79 ^(a)^	CATTTCATCTGTGAGCAAAGGTGG	64.4°	60°	24.146–24.169
**2R**	Primer forward CoV17 ^(b)^	ATCTCTGCTTTACTAATGTCTATGC	64.5°	60°	22.728–22.752
	Primer reverso 78 ^(a)^	TGTGTACAAAAACTGCCATATTGCA	64.5°	60°	23.823–23.847
**SEQ**	Primer foward 77 ^(a)^	CCAGCAACTGTTTGTGGACCTA	64.9°	60°	23.123–23.144
	Primer reverso 77 ^(a)^	CAGCCCCTATTAAACAGCCTGC	65.4°	60°	23.501–23.522

The name, sequence, melting temperature (T.M), annealing temperature (T.A), and position are shown. The terms in bold represent the steps of RT-PCR, where the first round (1R) will produce a 1645 bp strand and the second round (2R) will produce a 1120 bp strand. The sequencing of the strand generated by the 2R will allow the identification of 25 important target mutations. (a): removed primers from ARTICnetwork; (b): removed primers from Shaibu et al. [13].

**Table 2:** Mutations and possible variants identified by the protocol.

Lineage		Zeta	Gama	Delta	Omicron				Alfa	Beta	Lambda	Eta	Iota	Kappa	Epsilon	Mu
	Mutation	P.2	P.1	B.1.617.2	BA.1	BA.2	BA.4	BA.5	B.1.1.7	B.1.351	C.37	B.1.525	B.1.526	B.1.617.1	B.1.427	B.1.621
**K417N**					X	X	X	X		X						
**K417T**			X													
**N440K**					X	X	X	X								
**G446S**					X	X	X	X								
**L452Q**											X					
**L452R**				X			X	X						X	X	
**S477N**					X	X	X	X					X			
**T478K**				X	X	X	X	X								
**E484A**					X	X	X	X								
**E484K**		X	X							X		X	X			X
**E484Q**														X		
**F486V**							X	X								
**F490S**											X					
**Q493R**					X	X										
**G496S**					X											
**Q498R**					X	X	X	X								
**N501Y**			X		X	X	X	X	X	X						X
**Y505H**					X	X	X	X								
**T547K**					X											
**A570D**									X							
**D614G**		X	X	X	X	X	X	X	X	X	X	X	X	X	X	X
**H655Y**			X		X	X	X	X								
**Q677H**												X				
**N679K**					X	X	X	X								
**P681H**					X	X	X	X	X							X
**P681R**				X										X		
**A701V**										X			X			
**T716I**									X							

The set of these mutations is sufficient for the identification and differentiation of these 15 variants, represented in bold (zeta, gamma, delta, BA.1, BA.2, BA.4, BA.5, alpha, beta, lambda, eta, iota, kappa, epsilon, and mu), since some individual mutations are specific and each variant has a combination of mutations capable of differentiating them from each other. Four omicron sublineages can be differentiated due to the absence of two mutations, G496S and T547K (BA.2), and presence of L452R and F486V (BA.4/BA.5). The “X” marking in the body of the table represents the presence of mutations in each variant.

## Data Availability

The data presented in this study are available in Table S1 (Supplementary Materials).

## Supplementary Materials

The following supporting information can be downloaded at: https://www.mdpi.com/article/10.3390/diagnostics12112609/s1, Table S1: Sequence access number.
