# Supplementary material for: A Simplified Sanger Sequencing Method for Detection of Relevant SARS-CoV-2 Variants
Source: Diagnostics (Basel). 2022 Oct 27;12(11):2609. doi: 10.3390/diagnostics12112609 (PMC9689870; doi:10.3390/diagnostics12112609)
Supplement: Supplementary file 1 [file diagnostics-12-02609-s001.zip › Supplementery Material.pdf]

**Table S1.** Accession IDs of 103 partial sequences of the S gene, obtained from infected patients attended at the Professor Edgard Santos University Hospital, Salvador, Brazil.

| SCOV - ID | NCBI GenBank Acession | GISAID Acession |
|---|---|---|
| hCoV-19/Brazil/BA-LAPI-SCV-375/2020 | OP185035 | EPI_ISL_14236298 |
| hCoV-19/Brazil/BA-LAPI-SCV-433/2020 | OP185036 | EPI_ISL_14241979 |
| hCoV-19/Brazil/BA-LAPI-SCV-434/2020 | OP185037 | EPI_ISL_14242413 |
| hCoV-19/Brazil/BA-LAPI-SCV-464/2020 | OP185038 | EPI_ISL_14242828 |
| hCoV-19/Brazil/BA-LAPI-SCV-494/2020 | OP185039 | EPI_ISL_14242829 |
| hCoV-19/Brazil/BA-LAPI-SCV-608/2020 | OP185040 | EPI_ISL_14242893 |
| hCoV-19/Brazil/BA-LAPI-SCV-650/2020 | OP185041 | EPI_ISL_14243568 |
| hCoV-19/Brazil/BA-LAPI-SCV-679/2020 | OP185042 | EPI_ISL_14243586 |
| hCoV-19/Brazil/BA-LAPI-SCV-706/2020 | OP185043 | EPI_ISL_14243587 |
| hCoV-19/Brazil/BA-LAPI-SCV-727/2020 | OP185044 | EPI_ISL_14243588 |
| hCoV-19/Brazil/BA-LAPI-SCV-734/2020 | OP208035 | EPI_ISL_14244338 |
| hCoV-19/Brazil/BA-HUPES-SCV-746/2020 | OP185045 | EPI_ISL_14244334 |
| hCoV-19/Brazil/BA-HUPES-SCV-751/2020 | OP185046 | EPI_ISL_14244335 |
| hCoV-19/Brazil/BA-HUPES-SCV-757/2020 | OP185047 | EPI_ISL_14244336 |
| hCoV-19/Brazil/BA-HUPES-SCV-784/2020 | OP185048 | EPI_ISL_14244337 |
| hCoV-19/Brazil/BA-HUPES-SCV-813/2020 | OP208036 | EPI_ISL_14244304 |
| hCoV-19/Brazil/BA-HUPES-SCV-814/2020 | OP185049 | EPI_ISL_14244339 |
| hCoV-19/Brazil/BA-HUPES-SCV-821/2020 | OP185050 | EPI_ISL_14244340 |
| hCoV-19/Brazil/BA-HUPES-SCV-822/2020 | OP208037 | EPI_ISL_14244341 |
| hCoV-19/Brazil/BA-HUPES-SCV-830/2020 | OP185051 | EPI_ISL_14244342 |
| hCoV-19/Brazil/BA-HUPES-SCV-869/2020 | OP185052 | EPI_ISL_14244343 |
| hCoV-19/Brazil/BA-HUPES-SCV-918/2020 | OP185053 | EPI_ISL_14244560 |
| hCoV-19/Brazil/BA-HUPES-919/2020 | OP185054 | EPI_ISL_14250418 |
| hCoV-19/Brazil/BA-HUPES-S-924/2020 | OP185055 | EPI_ISL_14318065 |
| hCoV-19/Brazil/BA-HUPES-S-927/2020 | OP208038 | EPI_ISL_14318066 |
| hCoV-19/Brazil/BA-HUPES-S-997/2020 | OP185056 | EPI_ISL_14318113 |
| hCoV-19/Brazil/BA-HUPES-S1108/2020 | OP185057 | EPI_ISL_14318185 |
| hCoV-19/Brazil/BA-HUPES-S-1114/2020 | OP185058 | EPI_ISL_14318186 |
| hCoV-19/Brazil/BA-HUPES-S-1178/2020 | OP185059 | EPI_ISL_14318397 |
| hCoV-19/Brazil/BA-HUPES-S-1181/2020 | OP185060 | EPI_ISL_14318719 |
| hCoV-19/Brazil/BA-HUPES-S-1189-2/2020 | OP185061 | EPI_ISL_14318989 |
| hCoV-19/Brazil/BA-HUPES-S-1195/2020 | OP185062 | EPI_ISL_14319532 |
| hCoV-19/Brazil/BA-HUPES-S-1196/2020 | OP185063 | EPI_ISL_14319808 |
| hCoV-19/Brazil/BA-HUPES-S-1214/2020 | OP185064 | EPI_ISL_14319827 |
| hCoV-19/Brazil/BA-HUPES-S-1220/2020 | OP185065 | EPI_ISL_14319828 |
| hCoV-19/Brazil/BA-HUPES-S-1222/2020 | OP185066 | EPI_ISL_14319829 |
| hCoV-19/Brazil/BA-HUPES-SCV-1236/2020 | OP185067 | EPI_ISL_14429339 |
| hCoV-19/Brazil/BA-HUPES-SCV-1258/2020 | OP208039 | EPI_ISL_14429340 |
| hCoV-19/Brazil/BA-HUPES-SCV-1281/2021 | OP185093 | EPI_ISL_14436492 |
| hCoV-19/Brazil/BA-HUPES-SCV-1364/2021 | OP185094 | EPI_ISL_14436493 |
| hCoV-19/Brazil/BA-HUPES-SCV-1378/2021 | OP185095 | EPI_ISL_14436489 |
| hCoV-19/Brazil/BA-HUPES-SCV-1438/2021 | OP185096 | EPI_ISL_14436497 |
| hCoV-19/Brazil/BA-HUPES-SCV-1450/2021 | OP185097 | EPI_ISL_14436498 |
| hCoV-19/Brazil/BA-HUPES-SCV-1495/2021 | OP185098 | EPI_ISL_14436532 |

| | | |
|---|---|---|
| hCoV-19/Brazil/BA-HUPES-SCV-1496/2021 | OP185099 | EPI_ISL_14436531 |
| hCoV-19/Brazil/BA-HUPES-SCV-1497/2021 | OP185100 | EPI_ISL_14436494 |
| hCoV-19/Brazil/BA-HUPES-SCV-1548/2021 | OP208040 | EPI_ISL_14436496 |
| hCoV-19/Brazil/BA-HUPES-SCV-1556/2021 | OP185101 | EPI_ISL_14436491 |
| hCoV-19/Brazil/BA-HUPES-SCV-1587/2021 | OP185102 | EPI_ISL_14436530 |
| hCoV-19/Brazil/BA-HUPES-SCV-1595/2021 | OP185103 | EPI_ISL_14436499 |
| hCoV-19/Brazil/BA-HUPES-SCV-1600/2021 | OP185104 | EPI_ISL_14436490 |
| hCoV-19/Brazil/BA-HUPES-SCV-1627/2021 | OP185105 | EPI_ISL_14436529 |
| hCoV-19/Brazil/BA-HUPES-SCV-1649/2021 | OP185106 | EPI_ISL_14436528 |
| hCoV-19/Brazil/BA-HUPES-SCV-1654/2021 | OP185107 | EPI_ISL_14436527 |
| hCoV-19/Brazil/BA-HUPES-SCV-1655/2021 | OP185108 | EPI_ISL_14436526 |
| hCoV-19/Brazil/BA-HUPES-SCV-1695/2021 | OP185109 | EPI_ISL_14436525 |
| hCoV-19/Brazil/BA-HUPES-SCV-1756/2021 | OP185110 | EPI_ISL_14436524 |
| hCoV-19/Brazil/BA-HUPES-SCV-1826/2021 | OP185111 | EPI_ISL_14436495 |
| hCoV-19/Brazil/BA-HUPES-SCV-1828/2021 | OP185112 | EPI_ISL_14436523 |
| hCoV-19/Brazil/BA-HUPES-SCV-1855/2021 | OP185113 | EPI_ISL_14436522 |
| hCoV-19/Brazil/BA-HUPES-SCV-1880/2021 | OP185114 | EPI_ISL_14436521 |
| hCoV-19/Brazil/BA-HUPES-SCV-1889/2021 | OP185115 | EPI_ISL_14436520 |
| hCoV-19/Brazil/BA-HUPES-SCV-1956/2021 | OP185116 | EPI_ISL_14436519 |
| hCoV-19/Brazil/BA-HUPES-SCV-1980/2021 | OP208041 | EPI_ISL_14436518 |
| hCoV-19/Brazil/BA-HUPES-SCV-2018/2021 | OP185117 | EPI_ISL_14436517 |
| hCoV-19/Brazil/BA-HUPES-SCV-2055/2021 | OP185118 | EPI_ISL_14436516 |
| hCoV-19/Brazil/BA-HUPES-SCV-2056/2021 | OP185119 | EPI_ISL_14436515 |
| hCoV-19/Brazil/BA-HUPES-SCV-2115/2021 | OP185120 | EPI_ISL_14436514 |
| hCoV-19/Brazil/BA-HUPES-SCV-2154/2021 | OP185121 | EPI_ISL_14436513 |
| hCoV-19/Brazil/BA-HUPES-SCV-2195/2021 | OP185122 | EPI_ISL_14436512 |
| hCoV-19/Brazil/BA-HUPES-SCV-2213/2021 | OP185123 | EPI_ISL_14436511 |
| hCoV-19/Brazil/BA-HUPES-SCV-2255/2021 | OP185124 | EPI_ISL_14436510 |
| hCoV-19/Brazil/BA-HUPES-SCV-2368/2021 | OP185125 | EPI_ISL_14436509 |
| hCoV-19/Brazil/BA-HUPES-SCV-2438/2021 | OP185126 | EPI_ISL_14436508 |
| hCoV-19/Brazil/BA-HUPES-SCV-2444/2021 | OP185127 | EPI_ISL_14436507 |
| hCoV-19/Brazil/BA-HUPES-SCV-2469/2021 | OP185128 | EPI_ISL_14436506 |
| hCoV-19/Brazil/BA-HUPES-SCV-2474/2021 | OP185129 | EPI_ISL_14436505 |
| hCoV-19/Brazil/BA-HUPES-SCV-2523/2021 | OP185130 | EPI_ISL_14436504 |
| hCoV-19/Brazil/BA-HUPES-SCV-2534/2021 | OP185131 | EPI_ISL_14436503 |
| hCoV-19/Brazil/BA-HUPES-SCV-2604/2021 | OP185132 | EPI_ISL_14436502 |
| hCoV-19/Brazil/BA-HUPES-SCV-2731/2021 | OP185133 | EPI_ISL_14436501 |
| hCoV-19/Brazil/BA-HUPES-SCV-2732/2021 | OP185134 | EPI_ISL_14436500 |
| hCoV-19/Brazil/BA-HUPES-SCV-2806/2022 | OP185068 | EPI_ISL_14436552 |
| hCoV-19/Brazil/BA-HUPES-SCV-2816/2022 | OP185069 | EPI_ISL_14436551 |
| hCoV-19/Brazil/BA-HUPES-SCV-2841/2022 | OP185070 | EPI_ISL_14436550 |
| hCoV-19/Brazil/BA-HUPES-SCV-2842/2022 | OP185071 | EPI_ISL_14436549 |
| hCoV-19/Brazil/BA-HUPES-SCV-2844/2022 | OP185072 | EPI_ISL_14436548 |
| hCoV-19/Brazil/BA-HUPES-SCV-2846/2022 | OP185073 | EPI_ISL_14436547 |
| hCoV-19/Brazil/BA-HUPES-SCV-2904/2022 | OP185074 | EPI_ISL_14436546 |
| hCoV-19/Brazil/BA-HUPES-SCV-2922/2022 | OP185075 | EPI_ISL_14436545 |
| hCoV-19/Brazil/BA-HUPES-SCV-2950/2022 | OP185076 | EPI_ISL_14436544 |
| hCoV-19/Brazil/BA-HUPES-SCV-3107/2022 | OP185077 | EPI_ISL_14436543 |

| | | |
|---|---|---|
| hCoV-19/Brazil/BA-HUPES-SCV-3124/2022 | OP185078 | EPI_ISL_14436542 |
| hCoV-19/Brazil/BA-HUPES-SCV-3138/2022 | OP185079 | EPI_ISL_14436541 |
| hCoV-19/Brazil/BA-HUPES-SCV-3268/2022 | OP185080 | EPI_ISL_14436540 |
| hCoV-19/Brazil/BA-HUPES-SCV-3300/2022 | OP185081 | EPI_ISL_14436539 |
| hCoV-19/Brazil/BA-HUPES-SCV-3401/2022 | OP185082 | EPI_ISL_14436538 |
| hCoV-19/Brazil/BA-HUPES-SCV-3408/2022 | OP185083 | EPI_ISL_14436537 |
| hCoV-19/Brazil/BA-HUPES-SCV-3437/2022 | OP185084 | EPI_ISL_14436536 |
| hCoV-19/Brazil/BA-HUPES-SCV-3440/2022 | OP185085 | EPI_ISL_14436535 |
| hCoV-19/Brazil/BA-HUPES-SCV-3461/2022 | OP185086 | EPI_ISL_14436534 |
| hCoV-19/Brazil/BA-HUPES-SCV-3566/2022 | OP185087 | EPI_ISL_14436533 |
